# Exploring the impact of environmental, social, and governance on clean development mechanism implementation through an institutional approach

**DOI:** 10.3389/fpsyg.2022.890524

**Published:** 2022-08-25

**Authors:** Sue Kyoung Lee, Gayoung Choi, Taewoo Roh, So Young Lee, Dan-Bi Um

**Affiliations:** ^1^SK Forest, Seoul, South Korea; ^2^Green Technology Center, Seoul, South Korea; ^3^Global Business School, Soonchunhyang University, Asan, South Korea; ^4^Korea Advanced Institute of Science and Technology, College of Business, Seoul, South Korea; ^5^E.T.S. de Ingeniería Agronómi, Higher Technical School of Agronomic, Food and Biosystems Engineering, Technical University of Madrid, Madrid, Spain; ^6^Korea Maritime Institute, Busan, South Korea

**Keywords:** CDM implementation, CDM projects, ESG, institutional theory, sustainable development

## Abstract

The study hypothesizes that the environmental, social, and governance (ESG) of the host country have a significant effect on clean development mechanism (CDM) implementation. As CDM incorporates sustainable development as one of the objectives for the green transition, many countries endeavor to adopt and implement CDM as their cleaner production method. Based on the institutional theory, the study aims to investigate the mechanism by which the institutional process of each ESG pillar makes an opportunity for a host country and to see how such country-specific factors influence the implementation of CDM projects. A county-year unbalanced sample drawn from World Bank and multinational CDM project data was analyzed using panel logistic and Poisson regression. Panel regression results show that high-energy intensity and low renewable electricity output as an environmental pillar positively affect CDM implementation. Unemployment and undernourishment as a social pillar positively affect CDM whereas low government effectiveness and the high rule of law positively affect CDM. In the results of zero-inflated Poisson regression, the direction of government effectiveness was upturned. The findings have broadened and deepened the ESG pillar based on the institutional theory and emphasized sustainable development rather than economic outputs.

## Introduction

The impact of global warming has become an urgent issue worldwide, which severely impacts ecological and environmental well-being, and ongoing industrial development worsens this at an unprecedented pace (Ji et al., 2021a; Khan and Ozturk, 2021). Various efforts have been made to reduce greenhouse gas (GHG) emissions, the fundamental causes of global warming. Collaborative actions through various environmental and economic strategies are required to reduce GHG emissions (Wang R. et al., 2020; Wang Y. et al., 2020; Cheng T. et al., 2021; Cheng Y. et al., 2021; Ji et al., 2021b). It also includes cooperation between developed and developing countries. The United Nations Framework Convention on Climate Change (UNFCCC), adopted in 1992 and entered into force in 1994, was launched to promote international efforts to address the challenges caused by climate change. The initial implementation phase of the UNFCCC supports the concept of “common but differentiated responsibilities (CBDR).” (Castro, 2016) In other words, it places a greater burden on developed countries, recognizing that they are primarily responsible for historical emissions.

The Kyoto Protocol, adopted in 1997 and entered into force in 2005, is the first international regulation designed to implement the UNFCCC based on the concept of CBDR. The clean development mechanism (CDM) is one of the Kyoto mechanisms introduced through the Kyoto Protocol; Annex I countries called advanced countries under the UNFCCC can cost-effectively achieve their binding reduction goals. Developed countries support through capital and technology to carry out the projects to reduce GHG emissions in developing countries; the reductions generated from these projects are recognized as certified emission reductions (CERs) and can be used as domestic reductions. CERs generated can be traded between countries/companies. In other words, developing countries can sell CERs for reduced environmental pollutants to developed countries and thereby gain an opportunity to participate in the global carbon market and achieve sustainable development (Purohit, 2009; Lim and Lam, 2014; Zainuddin et al., 2017).

Clean development mechanism has started with the expectation that it will become a win-win system that benefits both developed and developing countries. CDM finances emission reduction projects with technologies needed for host countries, thereby contributing to low-carbon technology-related skills, employment, and capacity building for developing countries (Purohit and Michaelowa, 2008; Paulsson, 2009; Seres et al., 2009; CDM Policy Dialogue, 2012; Lim and Lam, 2014).

Contrary to the earlier implementation principles, the Paris Agreement and the United Nations 2030 Agenda for Sustainable Development, finalized in 2015, emphasize sustainable development visions through self-differentiation of countries’ responsibilities. To achieve this ultimate goal, the Paris Agreement provides countries in need of a framework for financial, technical, and capacity-building assistance. In other words, the implementation of the Paris Agreement is essential for achieving the sustainable development goals (SDGs) and provides the most comprehensive roadmap for climate actions that will reduce emissions and strengthen climate resilience. Previous studies found that climate actions outlined in the nationally determined contributions (NDCs) voluntarily submitted by each country to carry out the Paris Agreement promote synergies with national development priorities that reflect the 2030 Agenda (Junsheng et al., 2019; Zheng et al., 2019). In this regard, it is necessary to interpret CDM from a sustainable development point of view. In particular, sustainable development mechanism (SDM), a new carbon market mechanism for the new climate regime according to the Paris Agreement, further underscores the sustainable development of the country based on the existing CDM framework.

To address the above issues, we aim to explore the environmental, social, and governance (ESG) pillars at a country level that affects the implementation of the CDM based on a sustainable development perspective. Previous literature focuses on how factors of ESG at the firm level have affected CDM project acceptance, technology transfer, and environmental contribution (Kuo et al., 2021; Stefanoni and Voltes-Dorta, 2021). However, few studies bind the theoretical lens with ESG as a precondition for implementing CDM projects at a national level. In other words, although ESG competency at the national could suggest the possibility of winning an order for an eco-friendly national industry or project, existing studies emphasized the importance of each competency individually, which might lead to overlooking the exhaustive framework. Maignan (2001), Chapple and Moon (2005) tried to explain that national factors or national ideologies can explain the socially responsible activities of corporations. Ebrahimi and Koh (2021) combined institutional theory with product life-cycle thinking, serving as a sustainability decision assessment (Durana et al., 2021). However, factors that explain the social activities at the national level remain to be studied. This research is vital because when countries try to implement CDM projects, it cannot be successful without the host country’s reciprocal relationship and the ESG of the host country. Verifying the assumption that consideration of ESG can be adopted at the national level of CDM projects with an institutional perspective has high value as research on the ongoing carbon market.

By filling the above research gaps, our research has made three contributions to the literature on the implementation of CDM projects by ESG at the national level. First, based on the institutional theory, we extend the understanding that the environmental and social characteristics of CDM beneficiaries can provide cost-benefit opportunities for investment countries. Successful CDM projects continue to be copied and benchmarked through a mimetic isomorphism when additionality should be allowed. Second, our exhaustive approach provides a theoretical understanding of the contextual characteristics of each national ESG in a CDM project. Our findings suggest that interactions with stakeholders in the feasibility examination can act as an opportunity to strengthen the legitimacy of accepting and internalizing the norm for CDM projects in interacting with the environment rather than unilaterally. Third, this study presents a paradoxical perspective that the CDM project was originally designed to help the sustainability of least developed and small countries, but it may not. This finding suggests that remedies are needed by coordinating structural or substantive examination of the organizational body that manages CDM according to the country’s ESG situation.

## Theoretical background and hypothesis development

### Institutional theory

While various theories and frameworks have been used to explain the responses to climate change, institutional approach has been widely embraced, along with legitimacy theory and stakeholder theory (Pellegrino and Lodhia, 2012; Ortas et al., 2015; Bazo et al., 2019; Kitsis and Chen, 2021). These frameworks describe how companies maintain legitimacy to meet social expectations (Hrasky, 2011), report on GHG emissions to meet the information needs of stakeholders (Liesen et al., 2015), and determine the business strategies driven by institutional pressures (Aerts et al., 2006; Higgins and Larrinaga, 2014). In particular, institutional theory has been used to explain the external influence on an organization to move toward sustainability trajectories (Ioannou and Serafeim, 2012). The core of the theory is that the environment and social surroundings could significantly affect the development of formal structures within an organization exerting significant influence on the organization’s decision-making (Campbell et al., 1991; Campbell, 2007). The deeper features of social structure impact the norms, rules, and routines and act as the guidelines in an organization (Kauppi, 2013). In addition, institutional theory has explained corporate social responsibility (CSR) activities since CSR activities are shaped by social contexts and national systems and are influenced by general institutions and policies for engaging in socially responsible activities (Aguilera et al., 2007; Jackson and Apostolakou, 2010). Furthermore, recent studies applied institutional theory to explain ESG, providing empirical evidence on how different country-specific social and institutional schemes influence companies’ ESG performance (Ortas et al., 2015). In this study, we assume that since CDM as a representative method for responding to climate change has recently been widely implemented as a means of CSR and ESG activities, it is inevitable to regard CDM as an institutional instrument. CDM has incorporated sustainable development as one of its objectives, along with reducing GHG emissions. CDM is being implemented as a means of CSR (Johannsdottir et al., 2014; Benites-Lazaro and Mello-Théry, 2017).

From an institutional point of view, the existing literature related to the carbon project verified forest-based mitigation (Boyd et al., 2007), REDD+ (Peskett et al., 2011), and CDM (Alizadeh et al., 2014), respectively. Boyd et al. (2007) emphasized the importance of carbon finance as a potential policy strategy to address global climate change, deforestation, and social development in underdeveloped countries while focusing on the socioeconomic impact of forest-based mitigation projects that emerged under the United Nations Framework Convention on Climate Change. Taking Uganda as a sample, Peskett et al. (2011) looked into how chances for underprivileged rural producers or people close to the project are impacted by the changes in institutional arrangements related to the carbon finance portion of a project. Alizadeh et al. (2014) presented the case in Iran, where the implementation of the CDM project progressed improperly due to the factors such as a lack of adequate infrastructure and skilled professionals. Despite the exploratory approach of the existing literature, the discussion on how the theoretical mechanism of ESG at the national level attracts the CDM project remains in its infancy. Given that CDM must be founded on international cooperation before it can be used as a part of these activities, we suggest that it is an appropriate chance to examine CDM projects from an ESG perspective using institutional theory.

### Clean development mechanism project implementation

Clean development mechanism has started expecting that it would benefit both developed and developing countries. Developed countries can use CDM to minimize the relatively expensive domestic reduction burden and fulfill their reduction goals at a low cost through abroad projects. In contrast, underdeveloped countries can adopt it for transfer to boost national development. However, it is hard to measure the achievement of carbon offsetting since there is no agreed standard for evaluation. Also, the minor participation of local stakeholders or authorities makes the CDM lack transparency and accountability (Lövbrand et al., 2009; Kuchler, 2017), thus making the contribution to sustainable development vague. It shows that the CDM installation purpose of contributing to the sustainable development of developing countries is likely to be neglected in operating the CDM. Therefore, this study suggests that the CDM projects promoted so far from the ESG perspectives in implementing CDM projects integrate the needs and situation of the developing country and local authorities. Accordingly, the hypothesis corresponding to the three pillars, ESG, is as follows.

### Environmental determinants for clean development mechanism implementation

Clean development mechanism projects are more likely to invest in countries with a high-energy intensity than those with low-energy intensity to obtain more credits through projects. Of the proposed CDM projects from 2008 to 2012, the energy-intensive countries such as South Korea, India, Brazil, and China possessed the potential for green technology (Ellis et al., 2007). However, Bayer et al. (2013) verified the relationship between inflows of foreign direct investment (FDI) and CDM project implementation in China and found that FDI limits the implementation of CDM projects. Developed countries with FDI experiences have access to advanced technology, leading to significant productivity increases and lower-energy intensity and carbon dioxide emissions. Energy intensity is a concept that indicates how much economic output is produced with the same amount of energy, and if the level of technology increases and produces more economic output with the same amount of energy, energy intensity is considered low. In countries with low-energy intensity, the marginal cost for carbon emission reduction increases, and the profitability of CDM projects decreases (Saggi, 2002; Popp, 2011). Therefore, this study assumes that energy intensity and CDM projects have a positive relationship.

To earn more carbon credits through the project, CDM-investing countries are more inclined to participate in host countries with high renewable electricity generation opportunities. Pata (2018) conducted a study to verify the relationship between economic development and urbanization, renewable energy consumption, and CO2 emission in Turkey during 1974–2014. The results show that as urbanization progresses by 1%, CO2 emission per population increases by 0.272–0.482%, and when economic development goes by 1%, CO2 emission per population increases by 0.082–0.096%. However, although renewable energy consumption is considered one of the main CO2 emission reduction methods, it does not significantly affect CO2 emissions. This is because renewable energy consumption in Turkey accounted for only about 6.49% of total energy consumption. China is notorious for using a lot of fossil fuels worldwide, but at the same time, it actively promotes the use of renewable energy nationwide. As of 2019, China’s renewable electricity output has grown quite rapidly, accounting for about 27% of China’s electricity output. Given the rapid renewable electricity output growth rate, China is projected to peak its emissions in 2030 while achieving its carbon-neutral target by 2060 (IRENA, 2019; Gao et al., 2021; Li et al., 2021). This can be estimated that CO2 emissions will be effectively reduced when renewable electricity output is nationally active. Therefore, since investing countries trade CERs by reducing CO2 emissions in developing countries through CDM, CDM projects will be actively implemented if a host country’s renewable electricity output is high and CO2 emission reduction can be effectively achieved.

**H1.** The host country’s energy intensity will positively impact the CDM project.

**H2.** The host country’s renewable electricity output will negatively impact the CDM project.

### Social determinants for clean development mechanism

Unemployment of the unskilled population is a pivotal contributor to crime, political violence, and social backwardness. Income inequality generated through unemployment stimulates crimes while instilling a sense of relative deprivation in the low-income class (Mocan, 1999; Kelly, 2000; Kliestik et al., 2020; Valaskova et al., 2021a). However, CDM projects create opportunities for creating more jobs for the population. By assuming the co-benefits associated with logging residues for bioenergy production in East Texas, United States, Gan and Smith (2007)’s input–output modeling revealed that the most noticeable benefits of bioenergy production were income and job creation. Similarly, unemployment in the host country can help to meet the needs of the CDM project. Therefore, unemployment as a factor for investors’ social opportunity is expected to positively impact the CDM project’s activation for host countries.

The use of fossil fuels for producing goods and services is increasing worldwide (Koçak and Şarkgüneşi, 2018). Fossil fuel consumption releases greenhouse gases that contribute to climate change (Bilgili et al., 2016; Zhou et al., 2017; Čuljkovic, 2018), adversely affecting the poor in Asia and Africa. Poor households are dependent on ecosystem-based livelihoods and experience production loss due to several obstacles (e.g., climate change, temperature rise, different rainfall patterns, natural disasters, heat exposure, malnutrition, and disease transmission) to poverty eradication and sustainable economic development (Zhou et al., 2017; Valaskova et al., 2021b). From a long-term perspective, one way to respond to climate change is to convert existing industries into cleaner production. The increase in cleaner production helps to improve environmental pollution and solve the problem of poverty (Khan, 2021). CDM projects help to solve the host country’s environmental issues and improve the people’s household income. Carfora and Scandurra (2019) found that the annual income of rural residents increased by 5.75% through biomass-based CDM projects in rural China. Therefore, it can be assumed that countries with widespread poverty will be active in implementing the CDM project to solve climate change and poverty problems.

Countries with high poverty rates may prefer environmentally friendly technologies. Although developing countries do not have high-energy demand as industrialized countries, interest in growing crops for biofuel production by utilizing vast available arable land is growing. Through this, developing countries can expect job creation and income increase in rural areas, and advanced countries can respond to rising fossil fuel costs, instability of oil supplies, and climate change. Nevertheless, the pace of biofuel development in Sub-Saharan Africa is relatively low. This is because a significant proportion of Africa’s residents are net food buyers, and repurposing land used to produce food crops for biofuels could increase food security concerns and exacerbate poverty (Jumbe and Mkondiwa, 2013; Silva Filho et al., 2018). Therefore, in countries with extreme poverty, it can be interpreted that the food problem takes priority over benefits such as job creation and income increase. Based on this, this study assumes a positive relationship between the prevalence of undernourishment and the CDM project.

**H3.** The host country’s unemployment will positively impact the CDM project.

**H4.** The host country’s prevalence of undernourishment will positively impact the CDM project.

### Governance determinants for clean development mechanism

Governance is a multidimensional concept that can be divided into civic participation, political stability and absence of violence, government efficiency, regulatory quality, the rule of law, and corruption control. Civic participation refers to the freedom of expression and speech and the degree to which citizens can participate in elections, whereas political stability and the absence of violence refer to the likelihood that a government will be destabilized or overthrown by violent means. Government efficiency refers to the quality of public services and independence from political pressure, the quality of policy establishment and execution, and the reliability of government policy implementation. The rule of law refers to the level of trust and observance of social discipline by the elected, and corruption control refers to the extent to which public power is exercised for private gain (Halkos and Tzeremes, 2013).

Governance can influence the implementation of CDM due to a variety of factors. Environmental regulation is one such factor. According to the institutional theory, companies show that they care about legitimacy, image, and reputation to external stakeholders by complying with the system (Bansal and Hunter, 2003). Moreover, long-term growth may be hindered if a company does not meet institutional expectations (Teo et al., 2003). Therefore, firms are motivated to adopt practices that are assessed to be socially valuable to maintain legitimacy (Raza, 2020). Since these factors induce companies to adopt green management practices (Delmas and Toffel, 2004), an empirical finding that environmental regulation affects CDM supports this claim (Zainuddin et al., 2017).

Ucar and Staer (2020) examined that managers tend not to engage in anti-social behavior because the costs of violating social norms are high when corruption is absent; on the other hand, they tend to reduce pro-social behavior in highly corrupt environments. Corruption also reduces the rigor of government energy policies (Fredriksson et al., 2004) and weakens environmental regulation enforcement (Arminen and Menegaki, 2019). From the CDM investor’s point of view, severe institutional corruption hurts the project by incurring very high transaction costs for CDM investors and project executors (Phillips and Newell, 2013).

It is difficult to effectively promote low-carbon communities and respond to climate change through government policy. To further enhance the effectiveness of government policies, transparency in government policies is essential. The financial burden is significantly difficult to meet the GHG emissions reduction target. A country with low corruption can build trust and secure funding for climate change from the international community (Hasan et al., 2020). In addition, when economic policy uncertainty is high, firms become more conservative in their management because it is difficult to predict future cash flows and try to hold more cash (Li, 2019; Phan et al., 2019). In other words, when policy uncertainty is high, companies reduce eco-friendly activities to avoid cost concerns, uncertainties, and risks (Hou et al., 2022). As a result, uncertainty becomes an obstacle to committing their resources to low-carbon projects such as CDM (Hultman et al., 2012). Therefore, this study assumes that government effectiveness will positively affect the CDM project.

Welsch (2004) argues that a strict rule of law pressures companies to follow environmental policy guidelines, inducing companies to comply with pollution prevention protocols and reduce CO2 emissions. The strict rule of law, along with institutional capacity, is a factor that reduces uncertainty and creates a stable investment environment for CDM (Phillips and Newell, 2013).

**H5.** The host country’s governance effectiveness will positively impact the CDM project.

**H6.** The host country’s rule of law will positively impact the CDM project.

## Methodology

### Research context

Table 1 shows the cases of CDM implementation and non-implementation, the ratio of implementation and non-implementation, and the frequency of CDM implementation by years. The frequency of CDM implementation during the entire sample period increased sharply as of 2005 and then decreased significantly from 2012.

**TABLE 1:** Clean development mechanism implementations by year.

Year	CDM_No_	CDM_Yes_	CDM_Yes/Total_ (%)	CDM_Number_
2000	187	6	3.11	14
2001	187	6	3.11	36
2002	184	9	4.66	49
2003	178	15	7.77	67
2004	176	17	8.81	78
2005	171	22	11.40	78
2006	171	22	11.40	145
2007	164	29	15.03	275
2008	162	31	16.06	371
2009	160	33	17.10	499
2010	159	34	17.62	610
2011	159	34	17.62	607
2012	153	40	20.73	439
2013	154	39	20.21	246
2014	167	26	13.47	66
2015	175	18	9.33	29
2016	180	13	6.74	19
2017	183	10	5.18	20
2018	188	5	2.59	5
2019	191	2	1.04	2
Total		100	3655	

Table 2 shows CDM frequency in the top 10 countries where CDM was actively implemented at a 5-year interval during the sample period. China and India accounted for a significantly high proportion of all our countries, and CDM was gradually implemented until 2010. However, the frequency of CDM gradually tended to decrease from 2015.

**TABLE 2:** Top 10 countries’ CDM implementations by year.

Country	2000	2005	2010	2015	2019	Total
China	0	6	404	0	0	1716
India	8	27	85	10	0	817
Brazil	2	13	13	2	0	184
Mexico	0	7	2	0	0	90
Vietnam	0	0	18	0	1	78
Thailand	0	1	6	0	0	74
South Korea	0	0	16	0	1	65
Indonesia	0	2	12	1	0	60
Malaysia	1	1	11	0	0	59
Chile	0	2	3	1	0	45

**FIGURE 1:**
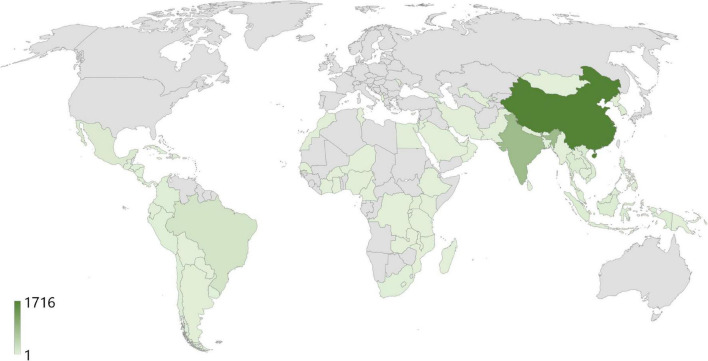
Clean development mechanism implementations by country (*N* = 3655).

Figure 1 is a visual representation of the results of Table 2. It was analyzed to determine the frequency of CDM implementations by regions and continents; the darker the green color in the figure is, the higher the frequency. The lowest CDM frequency recorded 1, whereas the highest reached 1,716, and approximately 80% of CDM was concentrated in Asia.

**FIGURE 2:**
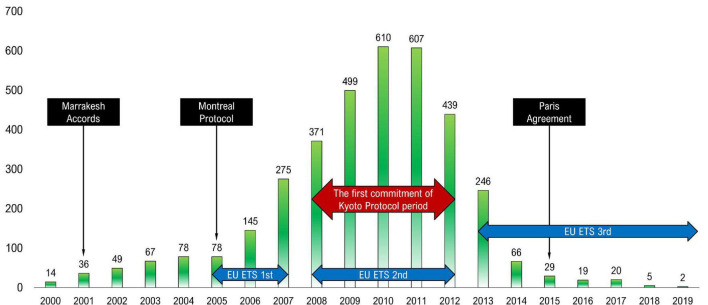
Institutional schemes over climate change and CDM implementations by year (*N* = 3655).

Figure 2 shows the changes in the institutional background at the time of CDM implementation fluctuation over the years mentioned above. The international community has been pouring in efforts by concluding various conventions for the ultimate efforts to respond to climate change through GHG reduction. For example, Marrakesh Accords was adopted during the Conference of the Parties (COP) 7 hosted in 2001. It led to the agreement on detailed rules for implementing the Kyoto Protocol, a climate change-related international convention. By officially adopting the Marrakesh Accords in COP11 in 2015, the Montreal protocol facilitated the implementation of the Kyoto Protocol (UNFCCC, 2021a). Moreover, as the first phase of the EU Emission Trading System (ETS), the international emissions trading system targeted most countries obligated to reduce GHG under the Kyoto Protocol, was carried out during 2005–2007, international activities for GHG reduction garnered attention (European Commission, 2021a). As the concrete carbon offset demand market activated, CDM adoption rapidly accelerated after 2005; CDM business rapidly increased during 2008–2012 when the Kyoto Protocol was first implemented, along with the 2nd phase of EU ETS (European Commission, 2021a,b).

However, despite the third phase of EU ETS during 2013–2020, CDM showed a declining trend as of 2012 (European Commission, 2021a) due to two main pillars. First, the decrease in CDM is caused by the SDM that will arrive as the next-generation global carbon market as a resolution of the UNFCCC COP. It was inferred that the CDM projects significantly decreased as the international community’s climate change measures to comply with the Paris Agreement shifted from CDM to SDM (Carbon Market Watch, 2017). In 2015, the Paris Agreement envisioned a framework for global climate change after 2020 and agreed to establish SDM. Thus, SDM is expected to grow based on CDM businesses and institutional foundations, in a form similar to that of CDM, as SDM aims to reduce GHG and pollutant emissions and sustainable development. Second, as EU-ETS entered the third phase, CERs were amended to be tradable in the market only when the CDM’s host country is an LDC, and carbon projects that clean HFC-23 and N2O, both of which are gray carbons, are not approved (ICAP, 2021).

### Sample and measurement

Table 3 shows the variables, definition/measurement, and reference used in this study. The dependent variables of this study are CDM_Yes_ and CDM_Number_, which refers to whether the CDM project was adopted or not, and the number of adoptions, respectively. This study developed variables by acquiring and analyzing CDM data by countries from 1999 to 2019 from UNFCCC CDM (UNFCCC, 2021a). CDM process consists of project design and national approval, validation, project registration, monitoring, verification and certification, and CERs issuance. Stages until project registration were regarded as *ex ante*, promoting GHG reduction activities, whereas the stages from which the previous stages’ result monitoring began were considered *ex post*. There are 15 sectors in the CDM, which are classified as large-scale and small-scale depending on the amount of GHG emission reduction and the project scale. Furthermore, CDM can be divided into Program CDM (PoA, Program of activities) and Project CDM, where the unit project constituting a program is classified as component of program activities (CPAs). For analysis, researchers in this study divided all CDMs into project units and analyzed them. Thus, if a country implemented the CDM project at least one time during a specific year, 1 was recorded in CDM_Yes_, and if not 0; CDM_Number_ represents the number of CDM implementations during the year.

**TABLE 3:** Definition/measurement and reference of variables.

Variables	Definition/Measurement	Ref.
*Dependent variables*		
CDM_Yes_	1: The country implemented at least one CDM in a given year 0: The country implemented no CDM in a given year	UNFCCC, 2021a
CDM_Number_	Number of CDM project activity by the country in a given year	UNFCCC, 2021a
*Control variables*		
Import	Imports of goods and services/GDP	World Bank WDI, 2021
Export	Imports of goods and services/GDP	World Bank WDI, 2021
Industry value added	Industry value (including construction)/GDP	World Bank WDI, 2021
CO2 emissions	Metric tons per capita	World Bank ESG, 2021
*Independent variables*		
Environment in ESG		
Energy intensity level	MJ/$2011 PPP GDP	World Bank ESG, 2021
Renewable electricity output	Renewable electricity/Total electricity output	World Bank ESG, 2021
Society in ESG		
Unemployment	Unemployment/Total labor force	World Bank ESG, 2021
Prevalence of undernourishment	Prevalence of undernourishment/Population	World Bank ESG, 2021
Governance in ESG		
Government effectiveness	Quality of public services, civil service, the degree of independence from political pressures, the quality of policy formulation and implementation, and the credibility of the government’s commitment to such policies	World Bank WGI, 2021
Rule of law	The extent to which agents have confidence in and abide by the rules of society, and in particular the quality of contract enforcement, property rights, the police, and the courts, as well as the likelihood of crime and violence	World Bank WGI, 2021

Data sources: World Bank ESG (https://databank.worldbank.org/source/environment-social-and-governance-(esg)-data), World Bank WDI (https://databank.worldbank.org/source/world-development-indicators), World Bank WGI (https://databank.worldbank.org/source/worldwide-governance-indicators), UNFCCC CDM (https://cdm.unfccc.int/Projects/index.html).

The first control variable in this study is export. This represents the value of services and goods sold to the global market. Export was measured through the exports of goods and services to GDP. Import was measured by the imports of goods and services to GDP. As the sum of values created by producers of all industries, excluding the value of intermediate goods and services from gross production, industry value added was measured by industry value (including construction) to GDP. As a by-product of fossil fuels, CO2 emissions were measured by annual metric tons per capita. Data for import, export, and industry value added were acquired from World Bank WDI (2021) and CO2 emissions data from World Bank ESG (2021).

Independent variables of this study are 6 different variables corresponding to E, S, and G. Variables under E are energy intensity level and renewable electricity output. The energy intensity level is the ratio of energy supply measured by purchasing power parity and GDP; it was measured by dividing the energy supply by GDP calculated in the value of USD in 2011. Renewable energy output is the power generated by renewable power generation and was measured by the ratio of renewable energy to the total annual power generation. All variables corresponding to E were acquired from World Bank ESG (2021). Variables under S are unemployment and prevalence of undernourishment. Unemployment is the proportion of the job-seeking labor force that is not working but can work. The prevalence of undernourishment refers to the proportion of all populations who do not eat enough food for a normal, active, and healthy life. All variables corresponding to S were obtained from World Bank ESG (2021). G consists of government effectiveness and the rule of law. Government effectiveness refers to efficiency, quality of public services, quality of public officials, independence from political pressure, quality of policy establishment and implementation, reliability in government policy, etc. The rule of law refers to “the perception of the possibility of agents abiding by social rules, contracts, property rights, police, courts, etc., and committing crimes and violence.” All variables under G were acquired from World Bank WGI (2021).

### Model estimations

We statistically test our theory using panel logistic regression. The model is specified as follows:


$$\mathrm{Pr}\left(y_{i\,t}\neq 0|X_{i\,t}\right)=P\,\left(X_{i\,t}\,\beta +v_{i}\right)$$

where *P* is the probability that country *i* will host the CDM project. Vector X_it_ represents the properties of country *i* (i.e., independent and control variables) in a given year. We partially corrected unobserved differences by adding a random-effect term in the random-effects model and excluding the time-invariant effect in the fixed-effects model (Greene, 2000). After examining the results of panel logistic analysis as random- and fixed-effects models, the Hausman Chi2 test was conducted for which model is more appropriate (Baltagi, 2021). In each model, vector X_it_ was t-1 lagged for the robustness check.

Our second estimation is panel Poisson regression using a fixed-effects model. For modeling count data, Poisson regression is frequently employed. There are a variety of adaptations to Poisson regression that are useful for count models. This model is often adopted when the dependent variable is a non-negative count. The model is specified as follows:


$$\mathrm{Pr}\left(Y_{i\,t}=y_{i\,t}|X_{i\,t}\right)=F\,\left(y_{i\,t},X_{i\,t}\,\beta +v_{i}\right)$$

where *Pr* is the probability that country *i* will host the number of CDM projects. The explanatory variables used are the vector X_it_ as in the panel logistic regression above. Since the fixed-effects model has already been proved to be more suitable for the model of this study than the random-effects model in panel logistic regression analysis, the fixed-effects model was used in our Poisson model with robust standard errors (Wooldridge, 1999). For the robustness check, vector X_it_ was t-1 lagged. In addition, we derived implications by splitting samples according to LDCs and SIDS using the fixed-effects Poisson model.

## Results

### Descriptive statistics

Table 4 shows the unbalanced pooled samples that integrated data from UNFCCC and World Bank and descriptive statistics of each variable. This study faced issues, including the absence of records of imports and exports in a specific year or country when extracting multiple years of data from different countries. As a result, the number of observations of each variable was uneven; thus, an unbalanced pooled sample was constructed.

**TABLE 4:** Descriptive statistics.

Variables	Obs.	Mean	*SD*	Min	Max
CDM_Yes_	3,860	0.11	0.31	0	1
CDM_Number_	3,860	0.95	12.11	0	404
Import	3,736	46.93	26.72	0.06	236.39
Export	3,736	40.81	27.98	0.10	228.99
Industry value added	3,863	26.75	12.30	3.15	87.80
CO2 emissions	3,800	4.37	5.41	0	47.70
Energy intensity level	3,139	6.63	5.20	1.09	43.35
Renewable electricity output	3,288	31.40	33.94	0	100
Unemployment	3,915	7.92	5.98	0.11	37.25
Prevalence of undernourishment	2,981	11.35	11.87	0.93	81.70
Government effectiveness	3,792	−0.07	0.99	−2.48	2.44
Rule of law	3,819	−0.06	1.02	−2.61	2.13

CDM_Yes_ recorded an average of 0.11 and a standard deviation of 0.31 for 3,860 observations. Response of CDM_Yes_ was only possible with 0 and 1, so the minimum value was 0, and the maximum value was 1. CDM_Number_ recorded an average of 0.95 and a standard deviation of 12.11 for 3,860 observations. Most of the coefficients were also significant at 0.05 in the correlation matrix. The variance inflation factor (VIF) range for all variables, including two dependent variables, was a minimum of 1.17 and a maximum of 3.69, confirming that there was less risk of multicollinearity (Hair et al., 1998).

### Panel logistic regression

Table 5 is the result of panel logistic regression, which was carried out for the data analysis of this study. Logistic regression analysis is an appropriate method for analyzing the relationship between the dependent variable and the independent variable in a non-linear relationship, such as whether CDM, the dependent variable of this study, is executed or not. Moreover, the Hausman specification test was conducted in this study to determine which random-effects (RE) model and the fixed-effects (FE) model were more suitable. When modeling panel data, the Hausman specification test is employed to determine whether a RE estimator uses time-invariant with constant value regardless of time or an FE estimator which does not use or reflect time-invariant as a dummy variant is suitable (Frondel and Vance, 2010). The Hausman specification test confirms the endogenous generation of time-invariant because bias may occur if the time-invariant is endogenous. FE can be considered more suitable because a significant value was derived from the Hausman specification test of this study. However, the RE model was also analyzed in this study to find out the difference from FE. Furthermore, Akaike information criteria (AIC) was utilized in this study for the suitability of the model. AIC score can determine the suitability of the model, where the model with the smallest score among other models after deriving scores using maximum log-likelihood from estimated parameters and models can be considered the most optimal (Salem and Salem, 2017; Zhang et al., 2020). In addition, FE_Lagged_ and RE_Lagged_ were used to see the effect when a 1-year lag was given to whether CDM was implemented.

**TABLE 5:** Panel logistic regression results.

Variables dependent variable: CDM_Yes_	Model 1	Model 2	Model 3	Model 4
	
	RE	FE	RE_Lagged_	FE_Lagged_
Import	0.006	0.018	0.020^+^	0.056**
	(0.013)	(0.020)	(0.012)	(0.020)
Export	−0.010	0.024	−0.016	0.001
	(0.015)	(0.021)	(0.014)	(0.021)
Industry value added	0.046*	−0.001	0.089***	0.108**
	(0.023)	(0.040)	(0.023)	(0.041)
CO2 emissions	−0.166*	0.160	−0.247**	0.006
	(0.072)	(0.139)	(0.077)	(0.151)
Energy intensity level	−0.116^+^	−0.178	−0.067	−0.132
	(0.068)	(0.118)	(0.062)	(0.102)
Renewable electricity output	0.001	−0.009	−0.005	−0.021
	(0.007)	(0.013)	(0.007)	(0.014)
Unemployment	−0.107**	0.003	−0.105**	0.000
	(0.036)	(0.056)	(0.037)	(0.057)
Prevalence of undernourishment	−0.075**	−0.095**	−0.038^+^	−0.020
	(0.024)	(0.032)	(0.022)	(0.031)
Government effectiveness	0.587	−0.012	0.352	−0.428
	(0.548)	(0.647)	(0.548)	(0.640)
Rule of law	−1.004^+^	0.250	−0.372	0.676
	(0.517)	(0.685)	(0.522)	(0.684)
Constant	−1.386		−3.191**	
	(0.934)		(0.979)	
Observations	1947	874	1947	874
Log-likelihood	−597.972	−338.662	−604.144	−339.435
Chi2	38.721	31.063	35.796	37.840
Prob > Chi2	0.000	0.001	0.000	0.000
AIC	1219.945	697.324	1232.288	698.870
Hausman Chi2 test	35.85**		41.11***	

RE, random-effect model; FE, fixed effect model; AIC, Akaike information criterion; standard errors in parentheses, ^+^*p* < 0.1, *p < 0.05, **p < 0.01, ***p < 0.001.

There are 1,947 observations for Model 1 and 3 and 874 observations for Model 2 and 4. Income, the control variable of this study, was found to be significant at the level of 0.1, 0.01 in Model 3 and Model 4, respectively. For industrial competitiveness, Model 1, Model 3, and Model 4 resulted in meaningful results at the 5, 0.1, and 1% levels, respectively. Finally, regarding CO2 emissions, Model 1 and Model 3 reached 5 and 1%, respectively, recording meaningful results.

The energy intensity level, an independent variable, showed partially meaningful results at the level of 10% in Model 1 regarding whether CDM was executed. As for unemployment, Models 1 and 3 showed statistical significance at the 1% level. Regarding the prevalence of undernourishment, results were derived from Model 1, Model 2, and Model 3 regarding the country’s CDM adoption. The rule of law shows that only Model 1 has a meaningful relationship at the 10% level regarding whether or not CDM is implemented.

However, there may be bias because relatively few countries with “Yes” were collected than countries with “No” CDM status. Therefore, since it may be difficult to analyze data only with logistic regression analysis, panel Poisson regression analysis was further conducted in this study.

### Panel Poisson regression

Table 6 represents the result of the Poisson regression analysis. The Poisson regression method is considered appropriate when the dependent variable is count data, such as the CDM_Number_, the dependent variable of this study. Based on the Hausman test in Table 5, FE was more suitable than RE, so FE was applied in Table 6, and the time lag was applied in Model 6.

**TABLE 6:** Panel Poisson regression results.

Variables	Model 5	Model 6
	
Dependent variable: CDM_Number_	FE	FE_Lagged_
Import	−0.031***	−0.050***
	(0.009)	(0.009)
Export	0.013	0.029***
	(0.008)	(0.008)
Industry value added	0.309***	0.368***
	(0.013)	(0.014)
CO2 emissions	0.495***	0.193***
	(0.038)	(0.036)
Energy intensity level	0.180***	0.209***
	(0.043)	(0.045)
Renewable electricity output	−0.024***	−0.025***
	(0.007)	(0.007)
Unemployment	0.051^+^	0.081**
	(0.027)	(0.026)
Prevalence of undernourishment	−0.224***	−0.190***
	(0.013)	(0.012)
Government effectiveness	−0.740***	0.197
	(0.195)	(0.201)
Rule of law	1.178***	2.019***
	(0.219)	(0.209)
Observations	902	902
Log-likelihood	−1633.068	−1754.975
Chi2	1208.747	1172.588
Prob > Chi2	0.000	0.000
AIC	3286.136	3529.951

FE, fixed effect model; AIC, Akaike information criterion, standard errors in parentheses, ^+^*p* < 0.1, **p < 0.01, ***p < 0.001.

There are 902 observations for CDM_Number_ in Table 6. Import, industry value-added, CO2 emissions, and the control variables reached meaningful 0.1% levels in both Models 5 and 6. Export was not statistically meaningful in Model 5 but highly significant in Model 6 (*p* < 0.001).

Energy intensity level, renewable electricity output, the prevalence of undernourishment, and the rule of law, the independent variables, all turned out to be significant in both Models 5 and 6 (*p* < 0.001). For unemployment, the coefficient was 0.051 in Model 5, partially statistically meaningful at 10% level, but in Model 6, the coefficient was 0.081, whereas the significant level was 1%, showing the differences among the models. For government effectiveness, a negative correlation with the coefficient of −0.750 was meaningful in Model 5 (*p* < 0.001), but the coefficient turned positive in Model 6, thereby losing its statistical significance.

Table 7 shows comparing LDCs and non-LDCs with panel Poisson regression. The dependent variable in Table 7 is CDM_Number_; FE and FE_Lagged_ were applied to Model 7 and Model 8 related to 734 non-LDCs; FE and FE_Lagged_ were applied to Model 9 and Model 10 related to 168 LDCs.

**TABLE 7:** Panel Poisson regression results with LDCs/non-LDCs.

Variables	Model 7	Model 8	Model 9	Model 10

**Dependent variable: CDM_Number_**	**Non-LDCs**		**LDCs**	
	**FE**	**FE_Lagged_**	**FE**	**FE_Lagged_**
Import	−0.037***	−0.057***	0.046	0.074*
	(0.009)	(0.009)	(0.038)	(0.037)
Export	0.016^+^	0.033***	0.039	−0.042
	(0.008)	(0.008)	(0.067)	(0.062)
Industry value added	0.316***	0.376***	−0.092	0.037
	(0.014)	(0.014)	(0.106)	(0.095)
CO2 emissions	0.505***	0.204***	2.563^+^	1.999
	(0.038)	(0.037)	(1.546)	(1.586)
Energy intensity level	0.192***	0.243***	−0.407	−0.500
	(0.045)	(0.046)	(0.321)	(0.305)
Renewable electricity output	−0.027***	−0.023**	0.006	−0.005
	(0.007)	(0.007)	(0.014)	(0.016)
Unemployment	0.053^+^	0.079**	−0.208	−0.047
	(0.027)	(0.027)	(0.227)	(0.214)
Prevalence of undernourishment	−0.230***	−0.198***	−0.072	−0.017
	(0.014)	(0.013)	(0.068)	(0.069)
Government effectiveness	−0.717***	0.249	−0.179	1.341
	(0.199)	(0.207)	(1.472)	(1.451)
Rule of law	1.165***	2.087***	0.025	−2.256
	(0.222)	(0.211)	(1.737)	(1.708)
Observations	734	734	168	168
Log-likelihood	−1554.118	−1662.542	−64.811	−69.716
Chi2	1202.962	1184.620	19.838	18.619
Prob > Chi2	0.000	0.000	0.031	0.045
AIC	3128.236	3345.084	149.623	159.431

LDC, least developed country; FE, fixed effect model; AIC, Akaike information criterion, standard errors in parentheses, ^+^*p* < 0.1, *p < 0.05, **p < 0.01, ***p < 0.001.

All control variables in Model 7 and Model 8 showed meaningful statistical results; exports reached 10% levels in Model 7, which was partially significant but reached 0.1% level with high statistical significance in Model 8.

In Model 7, all ESG variables, the independent variables, were meaningfully correlated with dependent variables, but the significance level of renewable electricity output dropped to 1% in Model 8. The coefficient value turned positive for government effectiveness but was insignificant. On the other hand, the significance level for unemployment stood at 1% in Model 8, having more statistical significance than Model 7.

On the contrary, in Models 9 and 10 related to LDCs, apart from the fact that import which is the control variable, reached 10% levels in Model 10, thereby being significant, whereas CO2 emissions turned out to be partially significant at 10% level in Model 9, all control variables turned out to be statistically insignificant. Also, all independent variables did not have a significant relationship with dependent variables. Thus, it is interpreted that ESG variables, including control variables, do not have a statistically significant effect on CDM in LDCs.

Table 8 shows the result of comparing SIDS and non-SIDs with panel Poisson regression. The dependent variable for Table 8 is the CDM_Number_; FE and FE_Lagged_ were applied to Model 11 and Model 12 related to 818 non-SIDS, whereas FE and FE_Lagged_ were applied to Model 13 and Model 14 regarding 84 SIDS.

**TABLE 8:** Panel Poisson regression results with SIDS/non-SIDS.

Variables	Model 11	Model 12	Model 13	Model 14

**Dependent variable: CDM_Number_**	**Non-SIDS**		**SIDS**	
	**FE**	**FE_Lagged_**	**FE**	**FE_Lagged_**
Import	−0.030**	−0.051***	−0.199^+^	−0.057
	(0.009)	(0.009)	(0.110)	(0.093)
Export	0.012	0.030***	0.234	0.110
	(0.008)	(0.008)	(0.143)	(0.120)
Industry value added	0.311***	0.372***	−0.812	−0.419
	(0.013)	(0.014)	(0.554)	(0.446)
CO2 emissions	0.497***	0.194***	1.897	2.157
	(0.038)	(0.036)	(1.846)	(1.962)
Energy intensity level	0.181***	0.209***	−0.568	−0.140
	(0.043)	(0.045)	(1.298)	(1.065)
Renewable electricity output	−0.025***	−0.026***	0.110	0.064
	(0.007)	(0.007)	(0.093)	(0.076)
Unemployment	0.054*	0.085**	−1.140	−0.651
	(0.027)	(0.027)	(0.707)	(0.627)
Prevalence of undernourishment	−0.224***	−0.191***	−0.385	−0.277
	(0.013)	(0.013)	(0.280)	(0.198)
Government effectiveness	−0.752***	0.180	−2.418	−1.642
	(0.196)	(0.204)	(3.615)	(3.253)
Rule of law	1.227***	2.106***	5.052	1.265
	(0.221)	(0.210)	(4.265)	(3.419)
Observations	818	818	84	84
Log-likelihood	−1602.419	−1717.516	−19.576	−25.048
Chi2	1210.773	1180.864	11.646	6.794
Prob > Chi2	0.000	0.000	0.309	0.745
AIC	3224.838	3455.032	59.152	70.096

SIDS, small island developing states; FE, fixed effect model; AIC, Akaike information criterion, standard errors in parentheses, ^+^*p* < 0.1, *p < 0.05, **p < 0.01, ***p < 0.001.

In Model 11, all control variables have a meaningful statistical correlation with the dependent variables. Export was insignificant in Model 11 but was statistically significant in Model 12 with a 0.1% level. As a result of verifying the relationship between ESG variables and CDM_Number_ in non-SIDS, the overall ESG variables were verified to have a statistically significant relationship with the CDM_Number_. Unemployment in Model 11 was significant at 5% and in Model 12 with 1%. The value for government effectiveness resulted in a negative correlation in Model 11 and turned positive in Model 12, although it was not statistically significant.

Except for import, control variables had no statistically significant correlations with dependent variables. In addition, significant statistical correlations between all independent variables and CDM_Number_ were not verified. Therefore, control and ESG variables did not affect CDM in LDCs and SIDS.

### Robustness check: zero-inflated Poisson with corrected Vuong regression

Poisson regression is commonly used when the dependent variable is count data (Liu et al., 2021). On the other hand, there is a possibility that the ratio of zeros increases by an additional mechanism that generates zeros in event-count processes, and verification through zero-inflated regression is preferred when the discrete data contain quite a lot of zeros (Desmarais and Harden, 2013; Yan et al., 2021).

**FIGURE 3:**
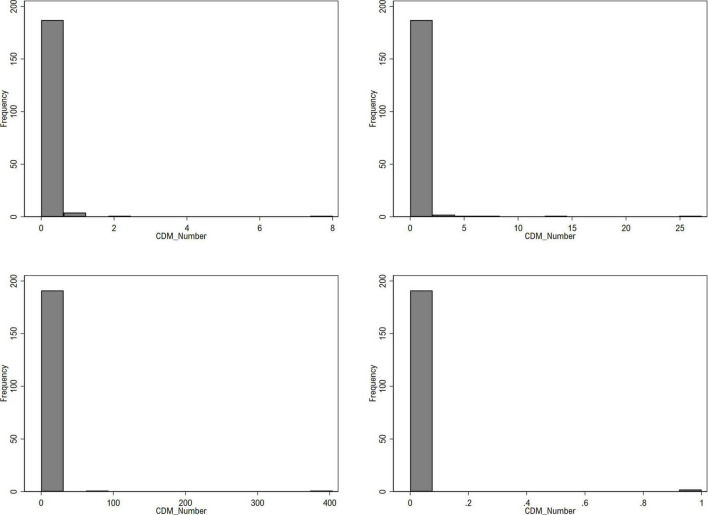
Histogram of CDM_Number_ by year 2000, 2005, 2010, and 2019 (from top left to bottom right).

Figure 3 shows the number of CDM projects in 2000, 2005, 2010, and 2019. In many countries, the number of CDM projects was 0, and a value other than 0 was exceptional.

Therefore, this study attempted to verify the robustness of our hypotheses through zero-inflated Poisson with corrected Vuong regression. The Vuong test is generally used to determine which zero-inflation component or single-equation count model is appropriate. Zero-inflated models contain more parameters than single-equation models, and the Vuong test can provide corrections for comparing models with different numbers of parameters (Vuong, 1989). When the result of the Vuong test added to the zero-inflated Poisson regression is significant, the zero-inflated model can be judged as more desirable. Vuong test result was statistically significant (*p* < 0.001).

In Table 9, all control and independent variables had a statistically significant relationship with the dependent variable. However, the difference from the panel Poisson regression result was that unemployment negatively affects the number of CDM, although the directions of the other independent variables were consistent. It can be interpreted as reflecting the characteristics of a country that has never implemented CDM to some extent.

**TABLE 9:** Results of zero-inflated Poisson with corrected Vuong.

Variables	Model 15	Model 16
	
Dependent variable: CDM_Number_	ZIPCV	ZIPCV_Lagged_
Energy intensity level	0.284***	0.271***
	(0.008)	(0.007)
Renewable electricity output	−0.011***	−0.011***
	(0.001)	(0.001)
Unemployment	−0.174***	−0.177***
	(0.008)	(0.009)
Prevalence of undernourishment	−0.024***	−0.031***
	(0.005)	(0.005)
Government effectiveness	0.967***	1.272***
	(0.113)	(0.116)
Rule of law	0.581***	0.443***
	(0.087)	(0.089)
Constant	0.678**	−0.682**
	(0.235)	(0.212)
Year dummies	Yes	Yes
Observations	1594	1594
Log-likelihood	−3111.543	−3063.320
Chi2	8298.517	8545.827
Prob > Chi2	0.000	0.000
AIC	6277.086	6180.641
Vuong statistics	6.504***	6.389***

ZIPCV, zero-inflated Poisson with corrected Vuong; AIC, Akaike information criterion, standard errors in parentheses, **p < 0.01, ***p < 0.001.

Table 10 summarizes the comparison between the predicted direction of each hypothesis in this study and the main test results. H1, H2, and H6 were consistently supported in all analysis results. Consistent with the panel Poisson model, H3 was supported, but the opposite result was derived in the zero-inflation model. It reflects the correction of errors in the characteristics of a country that has never implemented the CDM. H4 was rejected because it appeared opposite to the hypothesis. Although we predicted that H1, H2, H3, and H4 would all act as opportunities for host countries to implement CDM, it is interpreted that high poverty could play as a threat to investing countries. H5 was supported only in the zero-inflation model. It could be understood that the government’s efficient implementation of the CDM can operate as a barrier to having it even once experienced.

**TABLE 10:** Summary of hypothesis tests.

Hypothesis	Predicted direction	PP-FE	PP-FE_Lagged_	ZIPCV	ZIPCV_Lagged_
**(H1)** Energy intensity level	+	+	+	+	+
**(H2)** Renewable electricity output	−	−	−	−	−
**(H3)** Unemployment	+	+	+	−	−
**(H4)** Prevalence of undernourishment	+	−	−	−	−
**(H5)** Government effectiveness	+	−	n.s.	+	+
**(H6)** Rule of law	+	+	+	+	+

PP, panel Poisson; FE, fixed effects; ZIPCV, zero-inflated Poisson with corrected Vuong; n.s., non-significant.

## Conclusion and discussions

As the most representative technology transfer model of “GHG reduction” for climate change response, CDM has gone through continuous institutional supplementation based on numerous actual cases that took place during its long history. At the same time, it is expected to provide environmental-social benefits, including the environment and climate change response to both developed and developing countries (Stuchi Cruz et al., 2017). Through CDM, developed countries can ease the relatively high-cost domestic reduction burden and achieve the reduction goals with a comparatively low cost through overseas projects. Developed countries achieved technological transfer and additional capital investments through CDM, promoting national growth (UN, 2008). In this vein, it is forecasted that CDM can be utilized as an essential platform for green technology transfer in implementing the new climate regime according to the Paris Agreement (Benites-Lazaro and Mello-Théry, 2017).

Unlike the Kyoto Protocol, where GHG reduction obligations were only given to developed countries, the new climate regime under the Paris Agreement requires 165 countries, which account for about 96% of the world’s GHG emissions, to be obliged to reduce domestic GHG emissions through establishing national NDC (Liu and Feng, 2018). The UNFCCC COP26 mentioned that the role of the carbon market as a means for the parties to achieve their reduction goals would be further strengthened (UNFCCC, 2021b). In particular, based on the existing CDM, the aspect of voluntary cooperation and sustainability is suggested to be further highlighted under the new SDM framework, so the parties need to consider this in developing reduction businesses.

Sustainable development goals ultimately emphasize the need to tackle new global environmental issues, including developmental gaps, worsening inequality, climate change, and the international community’s solidarity to implement balanced growth considering the economic, social, and environment. Taking this into account, a carbon reduction strategy using CDM, which promotes GHG reduction under the new climate regime and sustainable growth, is even more vital as it can contribute to achieving both GHG reduction and SDG goals (Abdulrahman et al., 2015).

In this vein, this study was carried out to suggest ESG perspectives and examine the effects of each pillar as a host country-specific characteristic regarding the purpose of CDM, a global climate technology cooperation platform, to respond to market mechanisms. The theoretical and policy implications derived from this study are as follows.

### Theoretical contributions

This study viewed CDM from the perspective of ESG by applying institutional theory. The results of this study will be discussed in this section. First, from the perspective of institutional theory, environmental/social factors of CDM recipients can serve as an opportunity for investing countries regarding the cost-benefits. As a mimetic isomorphism in the institutional theory, the higher the energy intensity from an environmental perspective and the lower the renewable electricity output. The existing successful fields and national projects continue to be copied and benchmarked when CDM projects’ additionality should be acknowledged. This can also be linked to unemployment and the prevalence of undernourishment. More CDM businesses were carried out at times of higher unemployment rates and lower undernourishment rates, which shows that technology projects for GHG reduction were promoted by targeting countries with infrastructures relatively established at a certain level. In other words, from an investor’s perspective, business development is likely to take place focusing on business sectors where additionality may be acknowledged, mainly among emerging developing countries with economic development above a certain level. It is highly likely to be carried out by benchmarking the best practices performed by countries or companies of a similar status. It is in line with the fact that colleagues influence corporate CSR participation in the community discovered (Singh et al., 2021). Therefore, investors willing to start their businesses through CDM can find more practical solutions, ensure legitimacy, and succeed through existing cases by spending less through imitative actions (DiMaggio and Powell, 1983). In particular, the environmental uncertainty of the global carbon market will further strengthen this mechanism.

Second, from the governance perspective, it was expected that more CDM businesses would be carried out with higher government effectiveness and the rule of law. Nevertheless, our result shows that more CDM businesses were implemented with low government effectiveness and a high rule of law. It can be implied as a result of explaining minimum normative isomorphism. In other words, it might be a country with relatively low government efficiency, and CDM businesses are being carried out in countries with normative pressure in promoting CDM projects. In particular, validation by a third party in the CDM business might be a mechanism to strengthen it. Normative pressure in neo-institutionalism is a process in which organizations implicitly accept norms and internalize the language in interacting with the environment; instead, a unilateral and direct influence on the organization from the environment. The stakeholder consultation process amid the feasibility test should have served as an opportunity to strengthen them. The above results are consistent with the previous study (Daddi et al., 2020), which revealed that corporate climate sensitivity is affected by normative and imitative pressures. To distinguish their strategies, companies explore core “institutional” players, and imitative actions inspired by such experience of competitors are also demonstrated in the result of the climate change sector.

### Managerial contributions

The ESG perspectives as host country-specific characteristics are presented for the cause of CDM implementation, and each pillar’s analysis and consideration of the impact are as follows. First, from the environmental point of view, the higher the energy intensity and the lower the renewable electricity output, the more CDM that was expected to be implemented was consistently supported. CDM implementation centered on countries with relatively high-energy intensity, in other words, in developing countries pouring efforts to enhance their energy efficiency. Considering that Energy Intensity in Asian developing countries is improving at an annual rate of 3.3% (IEA, 2021), CDM might have been utilized as a tool for this. On the other hand, the renewable energy output is supported because countries with relatively low renewable energy ratios would have tried to increase their renewable energy ratio through CDM. CDM has indeed been contributing to reducing emissions since it was first implemented (UNFCCC, 2018); nevertheless, several limitations are also being pointed out (Kumazawa and Callaghan, 2012; Grunewald and Martinez-Zarzoso, 2016; Almer and Winkler, 2017; Maamoun, 2019). In particular, the most representative limitation of CDM business is that current CDM projects are not evenly carried out across all 15 fields but are concentrated in specific sectors, such as the power sector (UNFCCC, 2018). Such sector-biased phenomenon is caused by the need to carry out business in a stable manner and introduce technologies that can maximize the effect of GHG reduction from technology donor countries (investors) rather than the technology needs in developing countries (CDM Policy Dialogue, 2012). According to Peters and Geden (2017), investment decisions for carbon dioxide removal are made under deep uncertainty, capturing a combination of geopolitical uncertainties, technological uncertainties, and social acceptance. In other words, our results can reflect that the technology donor country promotes energy sector projects to maximize the emission reduction effect during technology transfer more than any other environmental factors when selecting a target country.

Second, it was expected that the higher the unemployment rate and undernourishment rate were from a social point of view, the more CDM implementations would be carried out. Nevertheless, the result showed that the higher the unemployment rate and the lower the undernourishment rate, the higher the frequency of CDM implementations. It can be inferred that technology projects for GHG reduction, such as CDM, have been promoted in countries with certain levels of infrastructure. The most critical factor in a CDM business is providing “additionality,” with the same recognition of “real” and “measurable” GHG reduction following the Kyoto Protocol Article 12 (Schneider, 2009). Additionality proves the additional occurrence of GHG reduction compared to baseline for the absence of CDM activities through CDM activities; it requires proving economic additionality apart from environmental additionality, GHG reduction (UNFCCC CDM, 2007). Economic additionality is a process of proving that unless investments take place through CDM, it is most unlikely for activities such as CDM to take place voluntarily (Schneider, 2009). To pass the validation in the CDM registration process of proving economic additionality, a specific CDM business should occur at a level where it does not generate “additional economic income.” The economic feasibility of just jumping over the hurdle of additional activity for GHG reduction must be proved. Therefore, what can be drawn from this result is that CDM is a business inevitably implemented in countries where job-seeking activities are actively taking place and in countries at the level of an “emerging” state, which has moved beyond the hunger state to a certain extent.

Third, from the governance perspective, it was expected that the higher the government of effectiveness, along with the rule of law, the more CDM projects were to be implemented. However, the result demonstrated that the lower the government effectiveness, the higher the rule of law, and the more CDM implementations took place. It can be perceived that traits of CDM projects have been reflected in the results. Once CDM successfully passes the validation process, the CDM is officially registered as a carbon offset business under the UNFCCC. The process of officially confirming the project approval of a host country is mandatorily required in this validation process; CDM project approval is issued by government organizations designated as Nationally Determined Authority (NDA) according to the UNFCCC regulations. According to the result, the lower the government of effectiveness, the more CDM implementations occurred; this demonstrates SDM’s opportunity of functioning as a mechanism complementing political uncertainty as well as government efficiency of the host country.

### Limitations and future studies

First, it should be possible to analyze the extent to which CERs are obtained through the CDM project and quantitatively analyze how much the results contributed to the GHG reduction of investing countries/corporates and developing countries. Second, a study on indicator development for sustainability is required. For example, CDM mandates the consideration of understanding and participation of residents as the direct beneficiaries of carbon reduction activities; however, verification has not been conducted closely due to a lack of data to verify this area. Thus, indicators on unemployment and undernourishment rates were inevitably used in this study as social factors. Still, indicators that can measure social impacts more accurately in the future should be developed through comparative research. Third, although the CDM projects are mutually reciprocal activities occurring in the relationship between developers and beneficiaries, studies on incentives that can be derived from CDM have not been conducted in various aspects. It is expected that if quantitative analysis of the CDM sector is possible, it will help to complement the limitations of the current research from an ESG perspective. In addition, looking at the purpose and interests of corporates carrying out individual CDM projects, it is hoped that they will be able to make more theoretical contributions in connection with CSR research. Furthermore, hopefully, the limitations of CDM will be supplemented, including the inconsideration of specific technology demands of development, regional bias, and the absence of technology business diversification. Finally, we expect the CDM-based SDM projects to become more prevalent as the role of the international carbon market is expected to attract more attention in achieving carbon neutrality. However, for SDM projects to succeed, it will be necessary to clearly understand the success/failure factors of the CDM project that has been promoted for the past 20 years. Based on this backdrop, the global climate consensus will accelerate and facilitate more efficient technology projects from an ESG point of view.

## Data availability statement

The original contributions presented in this study are included in the article/supplementary material, further inquiries can be directed to the corresponding author.

## Author contributions

TR supervised the data, did the conceptualization, performed the methodology, carried out the formal analysis, investigated the data, wrote the original draft, and wrote, reviewed, and edited the manuscript. SKL and GC did the conceptualization, wrote the original draft, and wrote, reviewed, and edited the manuscript and contributed equally to this work as first authors. SYL and D-BU wrote the original draft and wrote, reviewed, and edited the manuscript. All authors contributed to the article and approved the submitted version.
